# A Real-Time Contactless Pulse Rate and Motion Status Monitoring System Based on Complexion Tracking

**DOI:** 10.3390/s17071490

**Published:** 2017-06-24

**Authors:** Yu-Chen Lin, Nai-Kuan Chou, Guan-You Lin, Meng-Han Li, Yuan-Hsiang Lin

**Affiliations:** 1 Department of Electronic and Computer Engineering, National Taiwan University of Science and Technology, 10607 Taipei, Taiwan; D10302014@mail.ntust.edu.tw (Y.-C.L.); B10102031@mail.ntust.edu.tw (G.-Y.L.); B10102216@mail.ntust.edu.tw (M.-H.L.); 2 Department of Surgery, National Taiwan University Hospital, 10002 Taipei, Taiwan; chounaikuan@yahoo.com.tw

**Keywords:** contactless pulse rate measurement, motion detection, complexion tracking, motion index, FPGA

## Abstract

Subject movement and a dark environment will increase the difficulty of image-based contactless pulse rate detection. In this paper, we detected the subject’s motion status based on complexion tracking and proposed a motion index (MI) to filter motion artifacts in order to increase pulse rate measurement accuracy. Additionally, we integrated the near infrared (NIR) LEDs with the adopted sensor and proposed an effective method to measure the pulse rate in a dark environment. To achieve real-time data processing, the proposed framework is constructed on a Field Programmable Gate Array (FPGA) platform. Next, the instant pulse rate and motion status are transmitted to a smartphone for remote monitoring. The experiment results showed the error of the pulse rate detection to be within −3.44 to +4.53 bpm under sufficient ambient light and −2.96 to + 4.24 bpm for night mode detection, when the moving speed is higher than 14.45 cm/s. These results demonstrate that the proposed method can improve the robustness of image-based contactless pulse rate detection despite subject movement and a dark environment.

## 1. Introduction

With the rapid growth of integrated circuit design and manufacture, commercial wearable devices such as smart bands and smart jackets are being widely used to assess human heart rates and motion status using embedded electrodes and integrated inertial motion unit (IMU) sensors [1,2]. However, these devices contain the potential risk of current leakage or causing discomfort when used for a long period of time [3]. People with sensitive skin or neonates with fragile epidermis may experience rashes or allergic reactions as a result of extended use [4]. Recently, these problems have been solved by replacing direct contact based sensors, with contactless sensors. This novel technique allows access to human vital signs without directly contacting human skin [5].

The contactless approach can be achieved by measuring photo-plethysmography (PPG) signals remotely. This process is based on an electro-optic technique for noninvasively measuring the tissue blood volume pulses (BVPs) in the vascular tissue bed underneath the skin [6]. Much vital information, such as blood pulse, blood pressure, blood oxygen saturation (SpO2), and cardiac disease, can be acquired by continuously diagnosing the PPG signal [7]. In early studies, researchers found that contactless pulse rate measurement can be exploited by detecting the variation of absorbed/reflected light from the skin surface. The induced color difference is caused by the hemoglobin in blood absorbing different amounts of light in reciprocity with the heartbeat. Such subtle changes can be identified with an ordinary, general-used camera under normal fluorescent illumination [8,9].

More recently, the rapid growth of portable imaging devices like smartphones and laptops has prompted a trend towards transforming conventional contact PPG (CPPG) to remote PPG (rPPG) measurement [10]. However, the rPPG signal queried from the variation of blood vessel volume in the skin surface is sensitive to the outside noises produced by changes of illumination, and motion artifacts [11]. To reduce the effects on the rPPG signal, state-of-the-art work is taking place to demonstrate multiple methods to enhance signal quality and to address motion artifacts [12,13,14]. However, current methods still have problems with real-time implementation and show limited improvement in dealing with severe subject movement.

In addition to heart rate measurement, motion detection provides another interesting perspective regarding personal health, for use in procedures such as estimating sleeping quality and monitoring human activity, etc. Current methods used to detect human activity mostly rely on wearable sensor attachments [15,16], which have the same drawbacks as contact electrodes, causing discomfort and inconvenience over time. Consequently, in this paper, we provide a contactless approach to detect human activities with an image sensor. Additionally, we categorize human activities into four states: stationary, mild movement, strong movement, and subject absence, based on complexion tracking. By classifying subject movements, we propose a motion index (MI) to improve the accuracy of contactless pulse rate measurement in dynamic situations.

In comparison with the earlier studies of contactless measurements [17], this paper focuses more on using motion detection algorithms to improve the robustness of contactless pulse rate and motion measurement. Furthermore, to overcome the illumination problem, we modified a camera with near infrared (NIR) LEDs to achieve better dark mode measurement. In consideration of the real-time application, a Field Programmable Gate Array (FPGA) SoC system was chosen to perform the proposed framework. In combination with Bluetooth communication technology, all the detected health information, comprising the instant pulse rate and motion status, can be transmitted to a smartphone for data display and collection in real-time.

## 2. Materials and Methods

**Figure 1:**
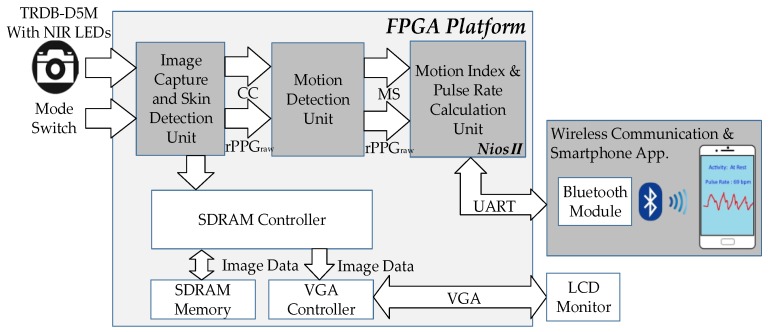
The architecture of the proposed system, which is developed based on an FPGA platform and a smartphone.

The proposed system architecture is shown in Figure 1, comprising an FPGA development board (DE2-115), a CMOS image sensor (TRDB-D5M), an HC05 Bluetooth module, and a smartphone. A mode switch is designed to alter the brightness and darkness mode detection. The proposed framework is constructed based on the hardware-software cooperative architectures that can be divided into three main parts: image capture and skin detection, motion detection, and motion index (MI) and pulse rate calculation. In the image capture and skin detection unit, the skin tone regions are determined through skin detection. The corresponding central coordinate (CC) and original rPPG are computed across each image frame. Subsequently, the obtained CC and the rPPG signal are transmitted to motion detection unit to determine the subject motion status (MS). In the pulse signal extraction and calculation unit, an indicator MI is used to identify the clean pulse signal. A few processing steps are introduced to compute the corresponding pulse rate. Finally, we have developed a smartphone application to receive the obtained pulse rate and motion status computed by the FPGA system through Bluetooth, to achieve a remote surveillance system.

**Figure 2:**
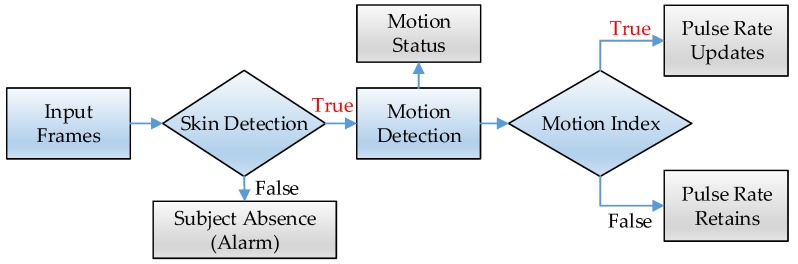
The data workflow of the proposed framework.

Figure 2 shows the data workflow of the proposed framework. The sequential image frames go through the skin detection algorithm. Frames containing less skin tone number than the setting threshold, are interpreted as indicating the subject is absent, and an alert signal is sent to the smartphone. Otherwise, the motion detection algorithm is performed to determine the subject’s motion status. To improve motion robustness, the motion index is proposed to preclude the distorted rPPG signal computation. The pulse rate is updated only when the motion index is true; otherwise, the pulse rate retains the same value from the last estimation. 

### 2.1. Image Capture and Skin Detection Unit

**Figure 3:**
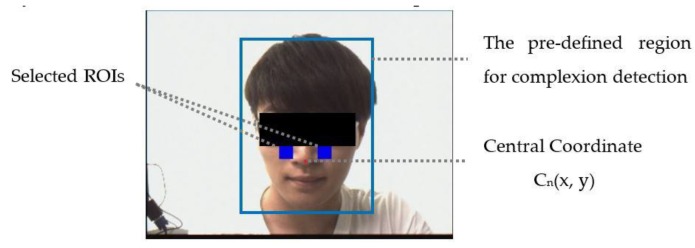
Illustration of the central coordinate and the region of interest (ROI) selection in both cheeks.

In this section, the serial of facial images is captured with a TRDB-D5M camera and transmitted to the FPGA development board at the frame rate speed of 22 fps, in RGB 640 × 480 format. Next, the skin detection and tracking method are performed to locate the skin tone region within the pre-defined area of the input frames, as seen in Figure 3. In this paper, we refer to the method provided in [18], which is able to detect multiple types of complexions extending from bright skin (RGB: 255, 229, 200), and intermediate skin to dark skin (RGB: 45, 34, 30). After the skin pixel is acquired, a scheme to check the subject existence (SE) is proposed. If the obtained complexion size is less than 5% of the size in the pre-defined region, the subject is defined as absent; otherwise, the subject is deemed present (1).

(1) $$\mathrm{SE}=\{\begin{array}{cc} \mathrm{False}, & P_{\mathrm{complexion}}<5\%\\ \mathrm{True}, & \mathrm{Otherwise} \end{array}$$

The principle of rPPG measurement is to sense the different reflectance caused by pulsation on the skin surface. Based on the vascular distribution, literature studies reveal that the cheek area contains the strongest pulsatile component for rPPG signal extraction [19]. Here, we compute the central coordinate of the captured skin tone pixel and adjust the relative region of interest (ROI) position to make them consistent on both sides of the cheek. The central coordinate (CC) is given by (2).

(2) $$C_{n}(x,y)=\frac{1}{N}\sum_{i=1}^{N}P_{i}(x,y)$$

where C_n_(x,y) refers to the central coordinate, N is the number of skin tone pixels within the pre-defined region, P_i_(x,y) are the location of the detected skin tone pixels, and n is current frame number.

Next, the relative position of the region of interest (ROI) is derived as two rectangles that are 30 pixels in width and 40 pixels in height at both sides of the cheek. Figure 3 also illustrates the selected ROI in this paper.

Once the ROIs are determined, the rPPG signal is acquired by computing the spatial mean of the proper color channel signal (green color channel for bright mode; red color channel for dark mode measurement) within the ROIs as follows (3) and (4) [14].

(3) $$S_{\mathrm{Right}/\mathrm{Left}}[n]=\sum_{i=1}^{30}\sum_{j=1}^{40}S_{n}(x_{i},y_{j})$$

where S_Right/Left_[n] is the sum of the green/red color signal within right/left ROI, S_n_(x_i_,y_j_) is the green/red color signal at location (x_i_,y_j_), n is the current frame number.

(4) $$\mathrm{rPP}G_{\mathrm{raw}}[n]=\frac{S_{\mathrm{Right}}[n]+S_{\mathrm{Left}}[n]}{{\mathrm{ROI}}_{\mathrm{total}}}$$

where S_Right_[n] and S_Left_[n] are the outputs of (3) at the right and left ROI, respectively, and ROI_total_ is the pixel size of two ROIs.

### 2.2. Motion Detection Unit

In this section, the novel approach to detect human motion status in a contactless way is interpreted. The motion status can be defined through measuring the displacement of the skin tone region across each amount of time. Generally, human movement is low frequency when compared to the camera’s capture speed. Therefore, the method that we proposed is to measure the displacement of the central coordinate every 0.5 s as follows (5).

(5) $$D_{n}=\sqrt{{\left(C_{n}(x)-C_{n-\tau }(x)\right)}^{2}+{\left(C_{n}(y)-C_{n-\tau }(y)\right)}^{2}}$$

where D_n_ is the displacement of the central coordinate at time n. C_n_(x) is the x coordinate of the computed centroid point. C_n_(y) is the y coordinate of the computed centroid point, τ is set as the unit of time, which is 0.5 s here.

After the displacement is determined, D_n_ is used to define the corresponding motion status (MS) as seen in (6).

(6) $$\mathrm{MS}=\{\begin{array}{cc} \mathrm{Stationary}, & D_{n}\leq \mathrm{Thres}h_{M}\\ \mathrm{Mild}\;\mathrm{movement}, & \mathrm{Thres}h_{M}<D_{n}\leq \mathrm{Thres}h_{L}\\ \mathrm{Strong}\;\mathrm{movement}, & D_{n}>\mathrm{Thres}h_{L} \end{array}$$

where Thresh_M_ and Thresh_L_ are the threshold values to differentiate the mild movement and strong movement, which are set to two and seven pixels respectively for the subject sitting at a distance around 50 cm from the camera.

Although we have reduced the measuring frequency, the obtained status is still too changeable and unstable for estimation every 0.5 s. In this section, we optimize the outcome by using a two second transition time. For each identical movement produced over 2 s, the MS is updated to the current status. Otherwise, the result remains as same as the previous estimation.

### 2.3. Motion Index and Pulse Rate Calculation Unit

In this section, a 32-bit embedded processor-Nios II is involved in functioning as the main processing unit to conduct the rPPG signal processing algorithm [20], since it provides a wide range of embedded computing applications and a friendly environment for development. 

On the basis of the previous studies, the contactless signal is sensitive to the subject movement and could reduce the performance for dynamic application [21]. To improve the pulse rate detection accuracy for dynamic measurement, we propose a motion index (MI) to prevent the failed motion affected signal ahead of rPPG signal processing. By referring to the status output in the motion detection unit, the MI value can be determined according to (7). 

(7) $$\mathrm{MI}=\{\begin{array}{l} 1,\;\mathrm{MS}=\mathrm{Stationary}\\ 0,\;\mathrm{MS}=\mathrm{Mild}\;\mathrm{movement}\\ 0,\;\mathrm{MS}=\mathrm{Strong}\;\mathrm{movement} \end{array}$$

After the MI is determined, the rPPG signal obtained from the ROI is passed through a bandpass filter, with the cutoff frequency set between 0.8 and 3.4 Hz (48~204 bpm). Subsequently, a 10-tap moving average is implemented to smooth the rPPG waveform. Additional processing details have been described in the previously published work [17]. 

Once the available pulse signal is obtained, the instant pulse rate is calculated by performing the real-time peak detection algorithm. In this paper, the instant peak detection with the adaptive threshold is utilized to locate the accurate peak occurrence by referring to [22]. The detected peaks are derived to the instant pulse rate by computing the mean of the time-lapse between two adjacent peaks (PPI) over a period of time. The pulse rate is converted into the generally expressed unit (beats per minute). The related operations are given in (8) and (9), respectively.

(8) $$\mathrm{PP}I_{\mathrm{avg}}=\frac{1}{N}(\sum_{i=n-N-1}^{n}\mathrm{PP}I_{i}),\mathrm{for}\;n\geq N$$

(9) $$PR_{n}=\frac{60}{\mathrm{PP}I_{\mathrm{avg}}}$$

where PPI_avg_ is the temporal mean of the two adjacent peaks over N PPI_s_. N is the averaged size referred from the ground truth device (N = 20 [23]). PPI_i_ denotes the interval between two adjacent peaks in second, and PR_n_ is the relevant pulse rate expressed in beats per minute (bpm).

### 2.4. Night Mode Detection

Another critical contribution of this paper is that we combined the night mode detection functionality in the proposed framework. Most of the presented works require sufficient lighting to extract the pulse signal by using an RGB color camera. Nevertheless, cardiac arrest or physical danger can occur during sleep or under conditions without adequate lighting. To overcome the illumination restriction, we modified the exploited D5M camera module with NIR LEDs. We also propose selecting the red color channel as the rPPG signal for better performance.

#### 2.4.1. Hardware Circuit

**Figure 4:**
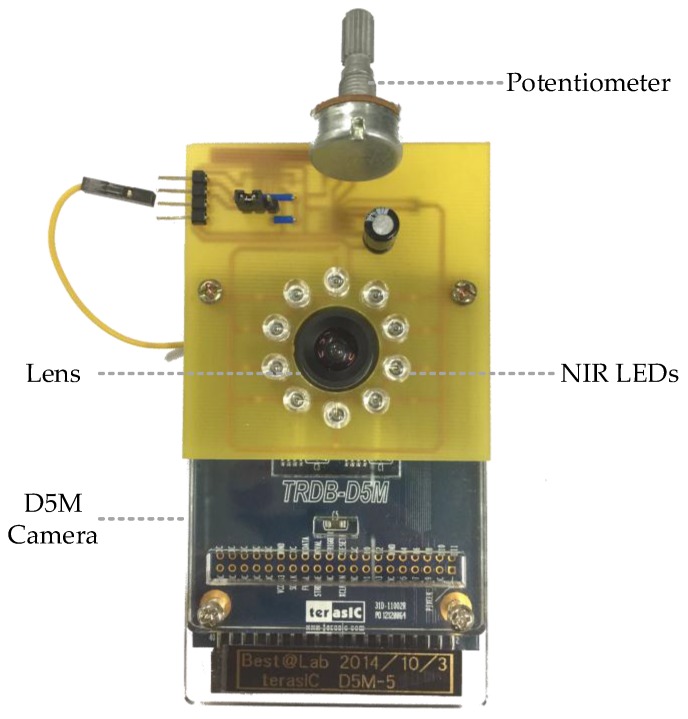
Modified camera with 10 add-on near infrared LEDs.

To achieve dark mode detection, many papers have proved the feasibility of using NIR LED (790~940 nm) to conduct pulse rate and SpO2 measurement [24,25]. To perform the method with the embedded system, we adopt 10 NIR LEDs (λ = 850 nm) to form a circular spotlight around the camera lens. The modified D5M camera can be seen in Figure 4, where the potentiometer is used to regulate the LEDs intensity.

#### 2.4.2. Color Channel Selection

The relevant works indicated that the rPPG signal produced by the green color channel has higher absorption variation and provides maximum PPG amplitude in the visible light wavelength [9]. However, we conducted a large scale experiment and found that performance degrades for signals registered in the green color channel in the NIR irradiation. Instead, the rPPG signal registered in the red color channel presents higher sensitivity for the vessel absorption variation. To make the system adapt to both ambient light and dark mode measurements, we design a General-purpose input/output (GPIO) switch for the proper color channel selection. The rPPG signal is captured from the red color signal when the NIR LEDs are on; otherwise, we use the green color signal as the default signal source.

## 3. Experiments and Results

In this section, we conduct a real-time experiment to evaluate the proposed system from several standpoints. First, the experimental setup is introduced, and the results are analyzed from several perspectives: the quantization of the movement categories, evaluating the accuracy of pulse rate detection and whether it involves the motion index (MI) implementation, and the performance comparison of selecting the red/green color channel as the rPPG signal in the dark mode detection.

### 3.1. Experimental Setup

**Figure 5:**
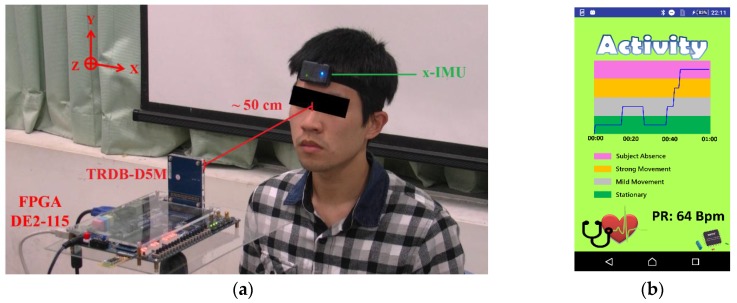
Experimental setup: (**a**) the system setup environment; (**b**) the developed android application for data display and collection.

**Figure 6:**
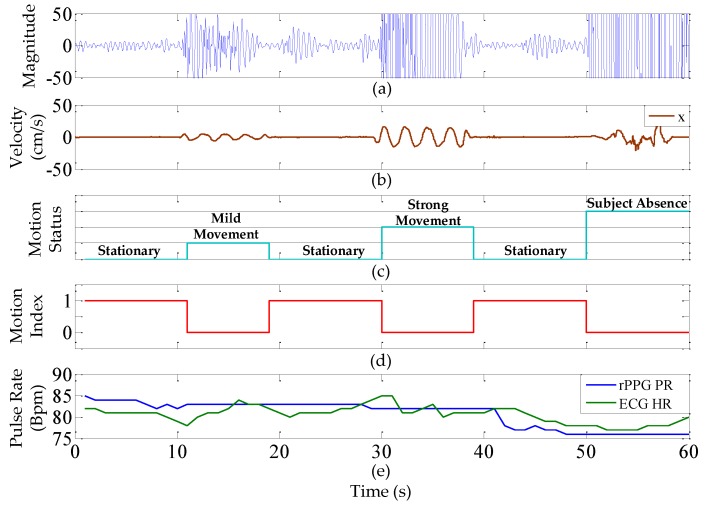
The data provided by one of the recruited subjects in ambient light measurement including: (**a**) the processed rPPG signal; (**b**) the velocity captured from the commercial inertial motion unit (IMU) device- (X-IMU) ; (**c**) the detected motion status; (**d**) derived motion index (MI); (**e**) rPPG pulse rate and ECG heart rate comparison.

We recruited ten volunteers (seven males, three females) in medium skin tone, and aged between 22 and 30 years old to participate in this study; volunteers had no cardiovascular diseases, as far as was known. The experimental environment is shown in Figure 5a; the subject sits in front of the proposed system at a distance around 50 cm with indoor fluorescent tubes (T5) as the only light source. The smartphone is connected to the system via Bluetooth to collect and display the detected pulse rate and plot the changes of motion status as shown in Figure 5b. To test the rPPG measurement motion robustness, the subject is requested to create several levels of movement in the following order: 10 s stationary, 10 s mild movement, 10 s stationary, 10 s randomly strong movement, 10 s stationary, and the last 10 s simulating subject absence by covering the face with the prepared blanket. In the experiment, a commercial inertial motion unit (IMU) device- (X-IMU) is attached to the subject’s front head to record the simultaneous motion signal [26]. The ground-truth ECG heart rates are collected with the validated chest strap [23]. Figure 6 shows the data from one of the subjects in the experiment.

### 3.2. Quantization and Movement Category

The baseline of each movement category is determined by integrating the accelerometer data collected from the IMU device. The average quantized value below 4.53 cm/s is categorized as stationary; 4.53 cm/s to 14.45 cm/s is categorized as mild movement activity; higher than 14.45 cm/s is categorized as strong movement.

### 3.3. Accuracy of Pulse Rate Detection

**Figure 7:**
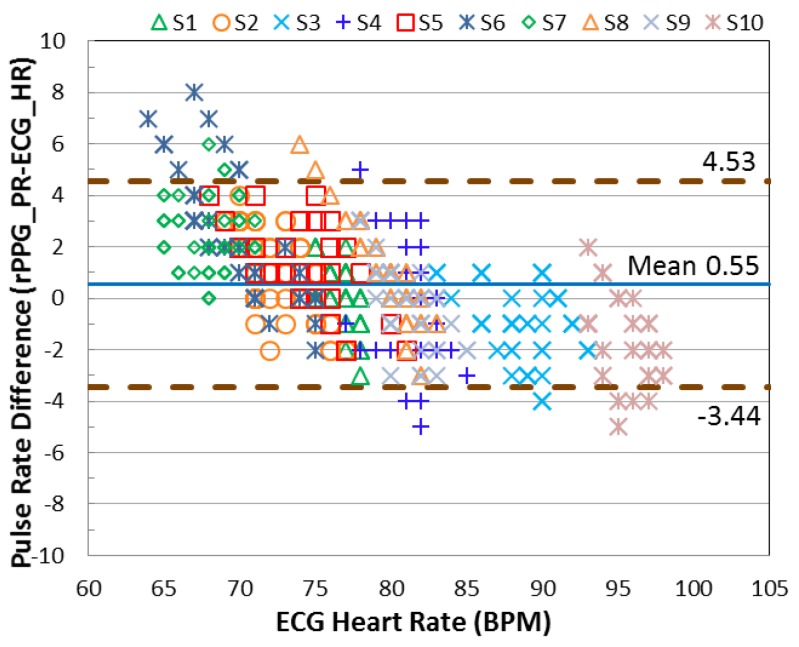
The Bland-Altman plot results of each subject creating three categories of movement; the processed method involves the proposed MI indicator.

**Figure 8:**
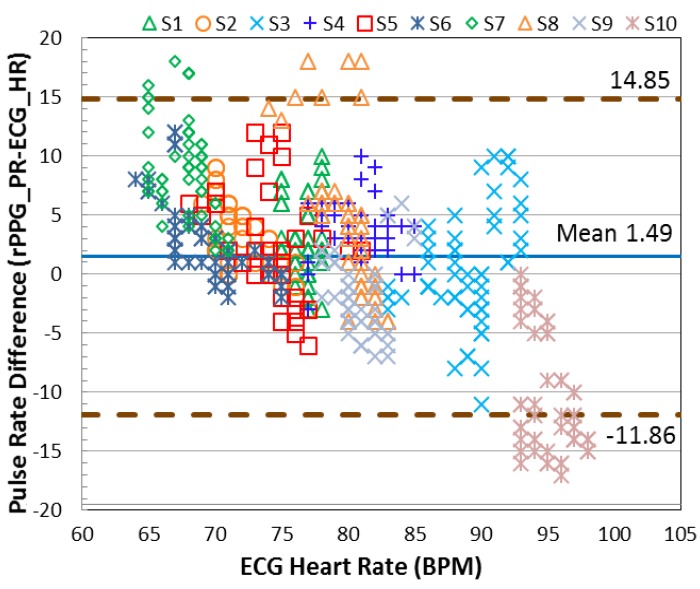
The Bland-Altman plot results of each subject creating three categories of movement, without the MI method.

In this section, the error of the detected pulse rate is analyzed according to three movement categories (stationary, mild movement, and strong movement). The standard deviation (SD) is computed individually and presented in Table 1. To validate the motion robustness of MI, the results are compared with the method without using motion index (MI). From the SD shown in the table, lower SD is provided when the MI is involved. To investigate the agreement between the detected pulse rate and the reference heart rate, the Bland-Altman plot is used to analyze the agreement between the detected pulse rate (rPPG) and the ground-truth heart rate (ECG). As seen in Figure 7 and Figure 8, the 95% limits of agreement ranging from −3.44 to +4.53 bpm are given by the MI applied system. Without using MI, the error expands from −11.86 to +14.85 bpm.

### 3.4. Accuracy of Night Mode Detection

To evaluate the proposed method of full darkness detection, six subjects are engaged to conduct the same experiment as Section 3.1. Only the different light source is provided by the exploited NIR LEDs instead. To ensure the subject is under the safe exposure level, we verify the irradiance with the radiometer (type: PM100D of Thorlabs Inc., New Jersey, NJ, USA). The value remains around 0.267 μW/m^2^ which is below the exposure safety limit of NIR.

**Figure 9:**
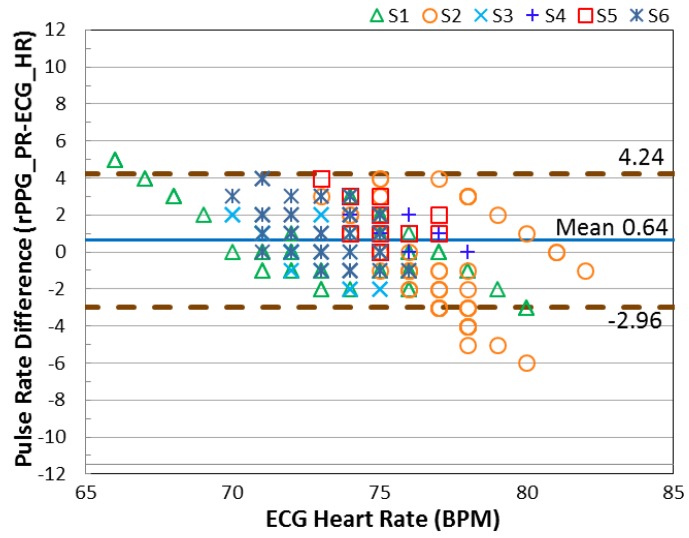
The Bland-Altman plot for the pulse signal registered in the red color channel under NIR LEDs illumination.

**Figure 10:**
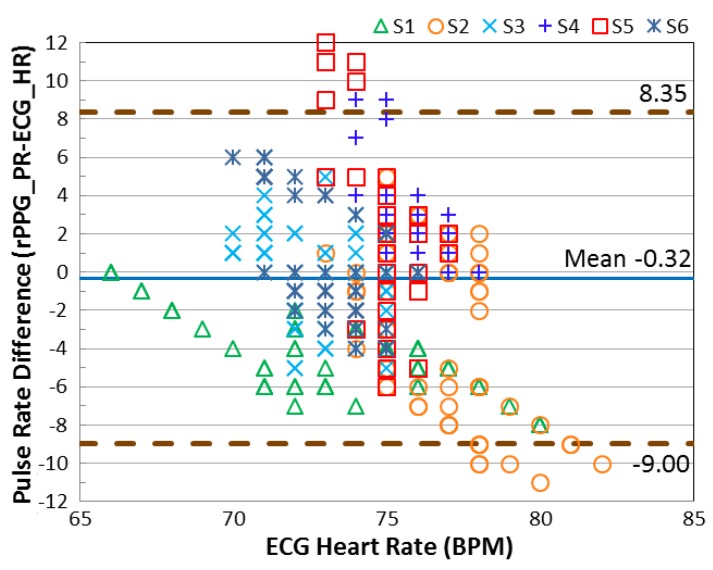
The Bland-Altman plot for the pulse signal registered in the green color channel under NIR LEDs illumination.

To investigate the performance of motion detection under NIR illumination, the results reveal that each movement category can be clearly differentiated with the proposed motion status detection. To demonstrate that the red color signal has better light absorption in the skin tissue under NIR radiance, we compared the performance of selecting the red color signal as the rPPG signal source with the green color signal. Both of the signals are processed with the proposed MI method and the identical processing algorithms. The results of the Bland-Altman plots in Figure 9 and Figure 10 show the better agreement for the signal registered in the red color channel, which ranges from −2.96 to +4.24 beats/min. In contrast, the agreement degrades for the rPPG signal accessing from the green color channel, which is between −9.00 and +8.35 beats/min when the NIR lighting was used.

## 4. Discussion

### 4.1. Application of the Contactless Motion Detection

The results demonstrate the proposed contactless system is able to monitor human motion status and pulse rate in both bright and dark conditions. On the basis of motion status monitoring, the proposed method appears to have multiple future applications such as surveillance for infant activity, long-term care system, and even for sleeping quality estimation. Additionally, the proposed subject existence checking scheme can be utilized to detect a patient off the bed situation. Furthermore, it could be used to detect objects blocking the face of an infant, which may cause dangers for an infant in sleep.

### 4.2. Pulse Rate Accuracy Evaluation with Motion Index

Recently, most of the related works focused on motion artifacts reduction by computing the linear combination in the RGB traces. In [14], the author adopted the signal registered in the red color channel to compensate for motion artifacts in the captured rPPG signal. Another effective method was proposed named Blood-volume pulse vector (PBV) in [27], indicating that the rPPG signal performed a unique signature (specific vector) in the normalized RGB color space. Consequently, the noise can be suppressed by conducting a linear combination of RGB signals based on the properties of the image sensor. Although these methods have proved the feasibility of addressing controllable motion artifacts, remotely detecting the induced color changes of pulsation still results in insoluble problems caused by serious image distortion (i.e., putting makeup on or unpredictable movements). These situations can conceal the induced pulsatile information that appears on the skin surface, which makes the signal barely extractable with the relevant works. Consequently, the proposed MI method in this paper is used to exclude the affected signal. Not only is the accuracy of pulse rate detection improved for the dynamic measurement, but also the complexity is reduced by reducing unnecessary computations.

### 4.3. Dark Mode Detection

As described in the result section, the pulse rate detection provides better performance when the red color channel is selected as the original rPPG signal source. The reason can be inferred from the spectral response of the D5M CMOS image sensor [28]. The datasheet shows the tendency of higher sensitivity in the red color channel for longer wavelength illumination. Although the spectrum in the invisible range is not provided in the datasheet, this study proves that the red color channel has better quantum efficiency in NIR illumination, according to our experiment.

### 4.4. Limitations of the Study and Future Works

The proposed system in this paper mainly focuses on providing a motion index indicator to improve the accuracy of contactless pulse rate detection in a dynamic situation and in dark measurement. For more accurate pulse detection, the current method adopted bandwidth of the bandpass filter between 0.8 and 3.4 Hz. Therefore, it cannot measure other vital signs such as the respiration rate in this condition. In the future, more vital signs derived from the contactless measurement including respiration rate, oxygen saturation, and blood pressure can be considered as future topics to increase the variety of measurements.

Although contactless measurement has been a new trend for allowing simpler and comfortable physiological signals measurement, the current developments are constrained and leave questions when facing various kinds of challenges, such as subjects with darker skin tones, or image distortion (i.e., makeup) measurements. In this paper, we mainly recruited subjects with medium skin tone and without wearing makeup or glasses to conduct the experiment. In the future, one of the objectives is to manage various skin tones and undertake different methods to improve the signal quality and robustness of the system.

## 5. Conclusions

In this paper, a real-time contactless pulse rate and motion status monitoring system based on an FPGA platform was designed and implemented. We proposed a motion index method to improve the accuracy of contactless pulse rate measurement when a subject creates several levels of movements. Experimental results of ten subjects show that the motion index can reduce the disturbance from the subject’s movement effectively, and narrow down the discrepancy from approximately ±14 to ±4 bpm. In addition, an effective method of choosing the red color channel as the rPPG signal source was proposed for higher accuracy of detection in a dark environment. We compared the results of selecting different color channels as data sources; the results indicate that the red color signal provided better sensitivity in the NIR environment and the pulse detection error can be mitigated from ±9 to ±4 bpm by choosing the red color signal. More importantly, we integrated the FPGA and smartphone platform to provide a robust monitoring system to not only measure the pulse rate, but also to monitor the motion status of the subject, which is the first step toward reliable and various monitoring solutions. In the future, more information can be provided with the contactless based system for healthcare and neonate surveillance applications.

## Figures and Tables

**Table 1:** The results of the pulse rate detection with/without MI implementation for all subjects in ambient light measurement.

Subject	Bias (bpm) with MI	SD with MI	Bias (bpm) without MI	SD without MI
Subject 1	0.17	1.07	2.47	3.11
Subject 2	1.05	1.52	2.67	2.75
Subject 3	−0.93	1.44	0.28	4.60
Subject 4	0.28	2.27	4.42	2.76
Subject 5	1.20	1.38	2.63	5.13
Subject 6	2.35	2.26	2.85	3.45
Subject 7	2.28	1.31	8.80	4.30
Subject 8	0.62	1.82	4.45	6.63
Subject 9	−0.02	1.50	−1.77	3.04
Subject 10	−1.52	1.57	−11.88	6.41
Average	0.55	1.61	1.49	4.22

## Abbreviation

PPG	Photoplethysmography
rPPG	remote PPG
CC	Central Coordinate
MS	Motion Status
MI	Motion Index
IMU	Inertial Motion Unit
